# The complete chloroplast genome of *Callicarpa siongsaiensis* Metcalf (Lamiaceae) from Fujian Province, China: genome structure and phylogenetic analysis

**DOI:** 10.1080/23802359.2021.1926364

**Published:** 2021-05-17

**Authors:** Yanqiu Xie, Site Luo, Linting Zhang, Hui Huang, Qun Zhang, Mingying Lai, Chuanyuan Deng

**Affiliations:** a College of Landscape Architecture, Fujian Agriculture and Forestry University, Fuzhou, Fujian, China; b School of Life Sciences, Xiamen University, Xiamen, China; c Island Research Center, Ministry of Natural Resources, Pingtan, Fujian, China; d Faculty of Architecture and Engineering, Fujian College of Water Conservancy and Electric Power, Sanming, China

**Keywords:** *Callicarpa siongsaiensis*, chloroplast genome, phylogenetic relationship

## Abstract

*Callicarpa siongsaiensis* Metcalf is a drought resistance shrub with ornamental potential. In this study, Illumina sequencing data were used to assemble the complete chloroplast genome of *Callicarpa siongsaiensis*. The length of the circular genome is 154,144 bp. It contains a total of 130 genes, including 87 protein-coding, 36 tRNA, and seven rRNA genes. The GC content of the chloroplast genome of *C. siongsaiensis* is 38.09%. The phylogenetic analysis fully resolved *C. siongsaiensis* in a clade with *C. formosana*.

*Callicarpa siongsaiensis* Metcalf is a shrub classified in the Lamiaceae, with high economic and ornamental value (Chen and Michael 1994). This species typically grows on rocky seaside slopes at an altitude of 20–100 m, and is native to Fujian, China (Chen and Michael 1994). *Callicarpa siongsaiensis* occupies a unique ecological niche, which makes it particularly vulnerable to the effects of climate change and habitat destruction. Morphologically, *C. siongsaiensis* is difficult to distinguish from other *Callicarpa* spp. (Tu et al.^2013^). Survey of the literature shows that no DNA sequences are published for *C. siongsaiensis*. In this study, we characterized the complete chloroplast genome sequence of *C. siongsaiensis* to serve as a genetic resource for future studies on the taxonomy of *Callicarpa*, and to get a better understanding of phylogenetic relationships in this genus.

The fresh leaves of *C. siongsaiensis* were collected from Pingtan Island, Fujian province, China (25°50′02″N,119.71′42″E). The voucher specimens of *C. siongsaiensis* was deposited at the Fujian Agriculture and Forestry University Herbarium (https://ysylxy.fafu.edu.cn/, Hui Huang, HuiHUANG@fafu.edu.cn) under the voucher number FZ-FJ2020-04A. The genomic DNA was extracted using Plant Genomic DNA Kit, DP305 (TIANGEN, Beijing, China). The sequencing library was produced using the Illumina Truseq™ DNA Sample Preparation Kit (Illumina, San Diego, CA) according to the manufacturer's recommendations. The prepared library was loaded on the Illumina Novaseq 6000 platform for PE 2 × 150 bp sequencing at Novogene (Beijing, China). The raw data were used to assemble the complete cp genome using GetOrganelle (Jin et al. 2020). Genome annotation was performed with PGA (Qu et al. 2019) by comparing the sequences with the cp genome of *C. formosana* Rolfe, GenBank accession number NC_052748 (Du et al. 2020).

The circular cp genome of *C. siongsaiensis* was 154,144 bp in length, containing a large single copy (LSC) region of 84,903 bp in length, a small single copy (SSC) region of 17,837 bp and two inverted repeats (IRs), each 25,702 bp. The total GC content is 38.09%, while the GC content of the LSC, SSC, and IRs regions is 36.20%, 32.29%, and 43.21%, respectively. A total of 130 unique genes were predicted, including 87 protein-coding, 36 tRNA, and seven rRNA genes.

A phylogenetic analysis was performed using complete cp genomes from 20 Labiatae species with *Fraxinus chiisanensis* and *Lysionotus pauciflorus* serving as the outgroup taxa. The genomes were aligned with the MAFFT v7.388 using default settings (Katoh and Standley 2013). The phylogenetic analysis was conducted based on maximum-likelihood (ML) analyses implemented in IQ-TREE v2.1.2 with the TVM + F+R5 nucleotide substitution model, which was selected by ModelFinder (Kalyaanamoorthy et al. 2017; Minh et al. 2020). The support for the inferred ML tree was inferred by bootstrapping with 1000 replicates. The analysis fully resolved *C. siongsaiensis* in a clade with *C. formosana* (Figure 1). This study provides important sequence information for species identification, and its phylogenetic relationship in the Lamiaceae.

**Figure 1.**
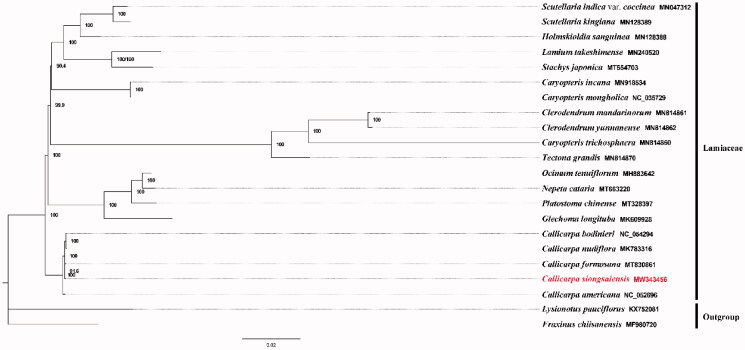
Maximum-likelihood (ML) tree based on 22 cp genome sequences of representative Labiatae. *L. pauciflorus* and *F. chiisanensis* were designated as outgroup. Numbers on the nodes are bootstrap values based on 1000 replicates. The *C. siongsaiensis* genome was marked in bold and red font.

## Data Availability

The genome sequence data that support the findings of this study are openly available in GenBank of NCBI at https://www.ncbi.nlm.nih.gov/ under the accession no. MW343456. The associated BioProject, SRA, and Bio-Sample numbers are PRJNA688996, SAMN17193101, and SRR13362732, respectively.
